# The efficacy and safety of knotless barbed sutures in total joint arthroplasty: a meta-analysis of randomized-controlled trials

**DOI:** 10.1007/s00402-018-2979-9

**Published:** 2018-06-16

**Authors:** Yanhong Han, Weiyi Yang, Jianke Pan, Lingfeng Zeng, Guihong Liang, Jiongtong Lin, Minghui Luo, Da Guo, Jun Liu

**Affiliations:** 1 Second School of Clinical Medicine, Guangzhou University of Chinese Medicine, Guangzhou, 510405 China; 2 Department of Orthopedics, Second Affiliated Hospital, Guangzhou University of Chinese Medicine, No. 111 Dade Road, Guangzhou, 510120 Guangdong China; 3 Bone and Joint Research Team of Degeneration and Injury, Guangdong Provincial Academy of Chinese Medical Sciences, Guangzhou, 510120 China

**Keywords:** Knotless barbed sutures, Total joint arthroplasties, Knotted traditional sutures, Randomized control trials, meta-analysis

## Abstract

**Background:**

The knotless barbed sutures (KBS) are an innovative type of suture that can accelerate the placement of sutures and eliminate knot tying. Whether the KBS are safe and efficient in total joint arthroplasty (TJA) remains controversial. Therefore, we conducted a meta-analysis to evaluate its efficacy and safety.

**Methods:**

Randomized-controlled trials (RCTs) were identified from the PubMed, Embase, and Cochrane Library databases up to October 2017. The Cochrane risk of bias tool was used to assess methodological quality. The statistical analysis was performed with RevMan 5.3.5 software.

**Results:**

A total of five RCTs (600 participants) were included in our meta-analysis. The results showed that KBS reduced wound suture time (MD − 4.51, 95% CI − 5.37 to − 3.66, *P* < 0.00001) and the wound suture cost (MD − 282.63, 95% CI − 445.32 to − 119.95, *P* < 0.00001), and did not significantly increase the rate of complications (OR 0.77, 95% CI 0.42–1.39, *P* = 0.13) or intraoperative events (OR 0.86, 95% CI 0.04–17.28, *P* = 0.92). There were no significant differences in ROM at postoperative 6 weeks and 3 months (MD − 0.74, 95% CI − 4.19 to 2.71, *P* = 0.67; MD − 0.30, 95% CI − 2.62 to 2.02, *P* = 0.80; respectively).

**Conclusion:**

Our findings suggest that KBS are a safe and effective method for TJA. Given the possible biases, adequately powered and better designed studies with longer follow-up are required to reach a firmer conclusion.

## Introduction

As the population ages and medical technology have improved, the rate of total joint arthroplasties (TJA) has increased considerably over the past decades. Total hip arthroplasty (THA) and total knee arthroplasty (TKA) are well-known popular surgical procedures for the treatment of degenerative disorders and traumatic diseases. This rapid growth in the number of surgeries has also coincided with innovations in surgical procedures that have improved postoperative function and minimized complications. However, improper soft-tissue handling remains a risk factor for complications after TJA. Secure wound closure is key to preventing infection, facilitating immediate rehabilitation, and improving the efficiency of TJA [1]. Therefore, sutures have received increasing attention in terms of innovation, as certain features can enable faster suturing and greater convenience in TJA. In addition, their quality is crucial for minimizing wound complications and withstanding force across the incision during the early postoperative joint motion [2].

The knotless barbed sutures (KBS) were first described by R.A. Mckenzie in 1967 [3], and the bidirectional barbed suture was first introduced in 2007 [1]. At present, KBS are used in several surgical specialties [4]. KBS has been demonstrated to provide better soft-tissue repair and shorter closure times than knotted traditional sutures (KTS) in urology, obstetrics, and plastic surgery [5–7]. However, whether KBS are efficient and safe in TJA remains controversial. Several studies have found that KBS offers several advantages, including shorter closure time, elimination of the need for knot tying and handling of multiple sutures, improved tissue distribution, and the use of less suture material [8–10]. Moreover, its postoperative clinical outcomes are similar to those of KTS [11–14]. Despite these potential advantages, barbed sutures are not commonly used in TJA. This might be due to their higher cost and uncertain clinical benefits. The previous research on the use of KBS in TJA is limited and has yielded conflicting results [15]. Some studies showed that barbed sutures were associated with more wound complications, whereas others found that they reduced closure time and costs [2, 11]. Campbell et al. found that KBS use was associated with a higher rate of infections requiring antibiotics than wound closure with KTS [15]. Furthermore, work of Smith et al. suggests that KBS are associated with a greater frequency and severity of wound-related complications [16].

Some meta-analyses have recently been conducted regarding the use of KBS in TJA. However, the outcomes of previous meta-analyses are unclear, and no studies have comprehensively examined the benefits of KBS. Consequently, no reliable evidence regarding which of the two suture methods (KBS or KTS) leads to better outcomes and lower rates of complications in TJA. Therefore, we performed a meta-analysis of published clinical research to assess the efficiency and safety of KBS in patients undergoing THA and TKA.

## Methods

### Search strategy

A comprehensive search of the PubMed, EMBASE, and Cochrane Library databases up to October 2017 was performed. The search terms included “knotless”, “barbed”, “arthroplasty, replacement, knee”, “total knee arthroplasty”, “total knee replacement”, “total knee prosthesis”, “arthroplasty, replacement, hip”, “total hip arthroplasty”, “total hip replacement”, and “total hip prosthesis”. The references of the identified studies were manually searched.

### Selection criteria

The inclusion criteria were as follows: (1) randomized-controlled trials (RCTs); (2) patients treated with primary total knee or hip arthroplasty; (3) knotless barbed sutures compared with knotted traditional suture; (4) data available for at least one of the key outcomes, including closure time, complications, closure cost, intraoperative events, Knee Society Score (KSS), or range of motion (ROM).

The exclusion criteria were as follows: (1) duplicate articles; (2) case reports, cohort studies, reviews, editorials, letters, and animal experimental studies; (3) data that could not be extracted.

### Data extraction

Two reviewers independently extracted the following data from the included studies: authors’ names, date of publication, sample size, patients’ age and gender, surgery type, closure time, complications, closure cost, follow-up periods, KSS, ROM, suture material, total closure time in the operating room, and intraoperative events. In the event of missing data, we attempted to contact the corresponding authors for details.

### Quality assessment

The methodological quality of the included studies was independently evaluated by two reviewers using the Cochrane Collaboration’s tool for assessing the risk of bias. These domains were selection bias (random sequence generation and allocation concealment), performance bias (blinding of participants and personnel), detection bias (blinding of outcome assessments), attrition bias (incomplete outcome data), reporting bias (selective reporting) and other bias (other sources of bias). Any disagreements were resolved by discussion or were arbitrated by the corresponding author.

### Statistical analysis

The meta-analysis was performed using Review Manager 5.3.5 (Cochrane Collaboration, Oxford, UK). Odds ratios (OR) and mean differences (MD) were used to pool dichotomous and continuous data, respectively. The pooled estimates regarding outcomes expressed as either dichotomous or continuous variables were calculated using the random-effects model (postoperative complications were assessed using the fixed-effects model). Heterogeneity was assessed using the Cochrane Q test and the I-square statistic. A sensitivity analysis was performed to identify the source of the heterogeneity. For all analyses, *P* < 0.05 was considered statistically significant.

## Results

### Study selection

**Fig. 1:**
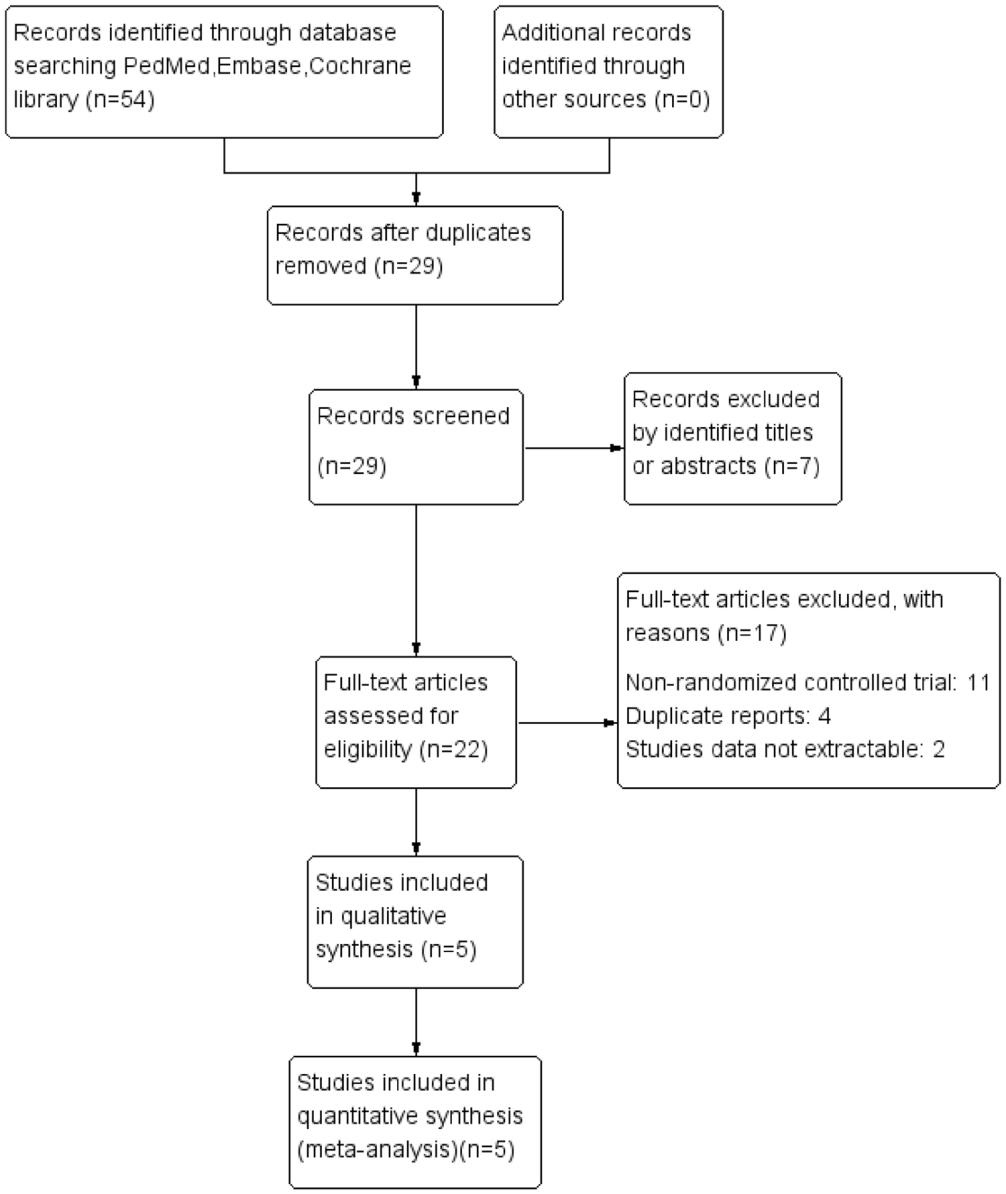
Flow diagram of identified, included, and excluded studies

A total of 29 records were identified via database and manual searches. After a thorough screening of titles and abstracts, seven records were excluded. The remaining 22 articles were assessed by full-text reviews. Finally, five studies met the inclusion criteria and were included in the meta-analysis (Fig. 1).

### Characteristics of the included studies

The characteristics of the included studies are presented in Table 1. Two studies involved TKA and THA [11, 16], and the remaining studies only involved TKA. The data set consisted of 600 participants, including 656 TKAs and 41 THAs. Among these, 345 participants’ wounds were closed with KBS and 352 with KTS. The gender ratio, average age, and types of surgery were also noted. In each study, the demographic characteristics of the two groups were similar.

**Table 1:** Characteristics of the included studies

Author	Date	Sample size		Gender (F/M)		Age (years)		BMI (kg/m^2^)		Knees/hips	Types of surgery	Follow-up periods (months)	Clinical outcomes
		KBS	KTS	KBS	KTS	KBS	KTS	KBS	KTS				
Chan^1^	2017	55	54	9/46	7/47	70.5 ± 8.2	70.4 ± 8.9	26.8 ± 1.2	26.5 ± 3.9	109/0	TKA	3	Closure time, complications, closure cost, ROM, KSS
Gililland^13^	2014	191	203	77/114	77/126	64 ± 10	63 ± 10	33 ± 8	33 ± 8	394/0	TKA	1.5	Closure time complications closure cost satisfaction score cosmesis score, KSS
Sah^2^	2015	50	50	21/29	21/29	68.1 ± 8.5	68.1 ± 8.5	30.1 ± 4.6	30.1 ± 4.6	100/0	TKA	12	Closure time, complications, closure cost, ROM, KSS
Smith^16^	2014	18	16	9/9	6/10	59.4(37 to 85)	64.3(24 to 86)	33.7(21.3 to 48.9)	30.1(22.7 to 44.4)	18/16	TKA & THA	NA	Closure time, complications, closure cost
Ting^11^	2012	31	29	23/8	21/8	64.4(41 to 86)	63.5(30 to 80)	30.4(20.5 to 45.5)	32.2(22.2 to 48.2)	35/25	TKA & THA	3	Closure time, complications, closure cost, satisfaction score cosmesis score
Total	–	345	352	139/206	132/220	–	–	–	–	656/41	–	–	–

*KBS* knotless barbed sutures, *KTS* knotted traditional sutures, *F* female, *M* male, *TKA* total knee arthroplasty, *THA* total hip arthroplasty, *ROM* range of motion, *KSS* Knee Society Score, *NA* data not available

For TKA and THA, details regarding the exact type of suture used, the relevant cost of suture material, and closure time are reported in Table 2. For surgeries involving both KBS and KTS, the placement and type of stitches varied. For surgeries using the KTS method, subcuticular tissue was closed with a running suture in three studies [1, 2, 16], while others used the interrupted suture technique. In the KBS group, the arthrotomy was closed with a running KBS in all studies.

**Table 2:** Suture type and cost of suture materials in the KBS and KTS groups

Author	Date	Suture materials compared		Average suture material costs		Total closure time in the operating room (minutes)		Total suture cost (mean ± SD)	
		KBS	KTS	KBS	KTS	KBS	KTS	KBS	KTS
Chan^1^	2017	Arthrotomy closure: 1-Stratafix Subcutaneous closure: 0-Stratafix Skin closure: surgical staples	Arthrotomy closure: 1-Vicryl Subcutaneous closure: 2/0 Vicryl Skin closure: surgical staples	$61.90	$14.50	10.52 ± 1.78	14.53 ± 3.16	$313.75±$42.61	$362.35±$75.65
Gililland^13^	2014	Arthrotomy closure: knotless #2 Quill SRS PDO Subdermal closure: knotless 0 Quill SRS Monoderm Skin closure: staples	Arthrotomy closure: interrupted #1 Ethibond Subdermal closure: 2-0 Monocryl Skin closure: staples	$24	$2	9.8 ± 4.2	14.4 ± 3.98	$324±$118	$419±$116
Sah^2^	2015	Retinaculum closure: 2 Quill™ Deep-intermediate layer closure: 2 − 0 Vicryl™ Subcutaneous closure: 2 − 0 Monoderm™ Subcuticular closure: 2 − 0 Monoderm™	Retinaculum closure: 1 Vicryl™ pop-off sutures Deep-intermediate layer closure: 2 − 0 Vicryl™ Subcutaneous closure: 2 − 0 Monocryl Subcuticular closure: 3 − 0 Monocryl	$82	$32	11.4 ± 2.2	16.1 ± 2.1	$307.6±$134.4	$804.8±$100.8
Smith^16^	2014	Fascia closure: #2 Quill Fat closure: #1 Quill Subcutaneous closure: #0 Quill Subcuticular closure: 2 − 0 Quill Monoderm	Fascia closure: #1 Ethibond Fat closure: 0-Vicryl Subcutaneous closure: 2.0 Vicryl Subcuticular closure: 3 − 0 Monocryl	$106.33	$14.40	16.78 ± 3.28	26.50 ± 6.83	$1213.8±$216.48	$1763.4±$450.78
Ting^11^	2012	Deep fascia closure: 2-polydioxanone Subcutaneous closure: 0-polydioxanone Subdermal closure: 2 − 0 monoderm Skin closure: skin staples and adhesive	Deep fascia closure: 1-Vicryl Subcutaneous closure: 0-Vicryl Subdermal closure: 2 − 0 monofilament Skin closure: skin staples and adhesive	$52.84	$9.43	9.2 ± 1.88	12.7 ± 3.08	$1000.44±$316.73	$1317.53±$193.13

*KBS* knotless barbed sutures, *KTS* knotted traditional sutures, *SD* standard deviation

### Risk of bias

**Fig. 2:**
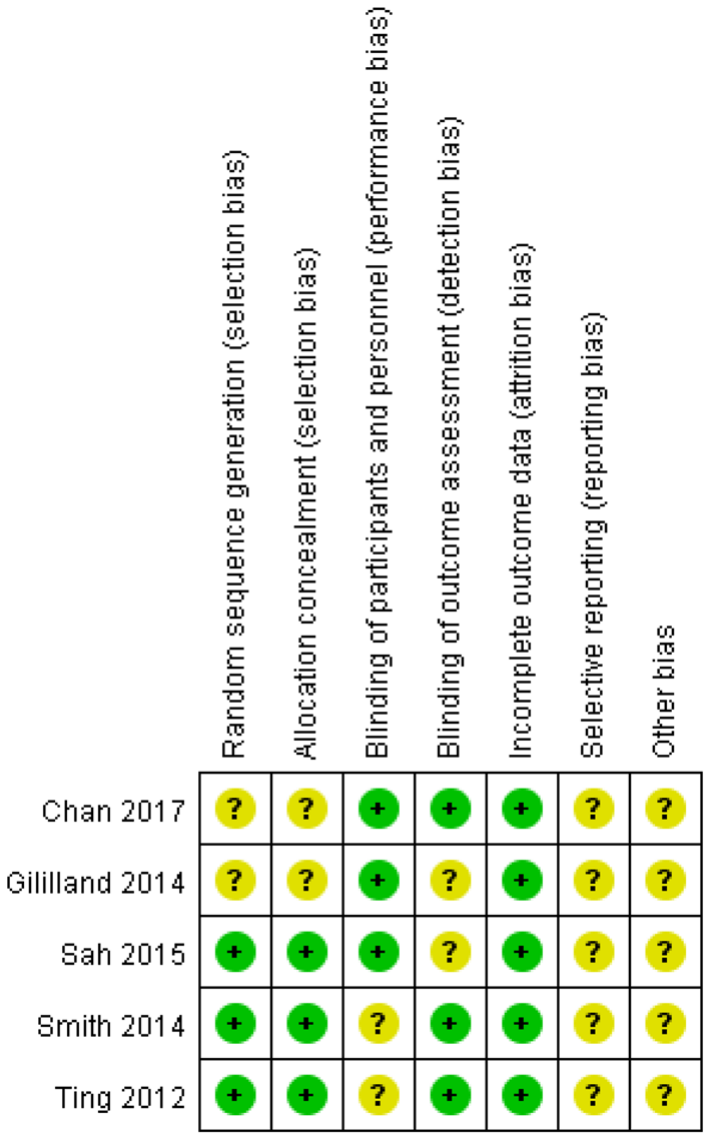
Summary of risk of bias for the included RCTs

The assessment of risk of bias is shown in Fig. 2. Random sequence generation was mentioned in all the included studies. Three of the studies detailed the methods of randomization [2, 11, 16]. Three studies described adequate allocation concealment [2, 11, 16]. All the studies mentioned the blinding methods: only one performed double-blinding of surgeons and assessors, and the others performed blinding of the study surgeons or assessors. None of the studies had a high risk of incomplete outcome data due to a lack of details regarding some adverse events. In addition, reporting bias and other bias were not described in any of the included studies.

### Wound closure time

**Fig. 3:**
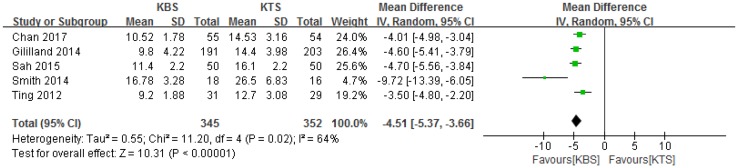
Forest plot and meta-analysis of wound closure time

All five studies reported the wound closure time [1, 2, 11, 13, 16]; subsequently, the data from these studies were pooled. The pooled results showed that KBS significantly reduced the wound closure time (MD − 4.51, 95% CI − 5.37 to − 3.66, *P* < 0.00001; Fig. 3), with moderate heterogeneity (*P* = 0.02, *I*^2^ = 64%).

### Complications

**Fig. 4:**
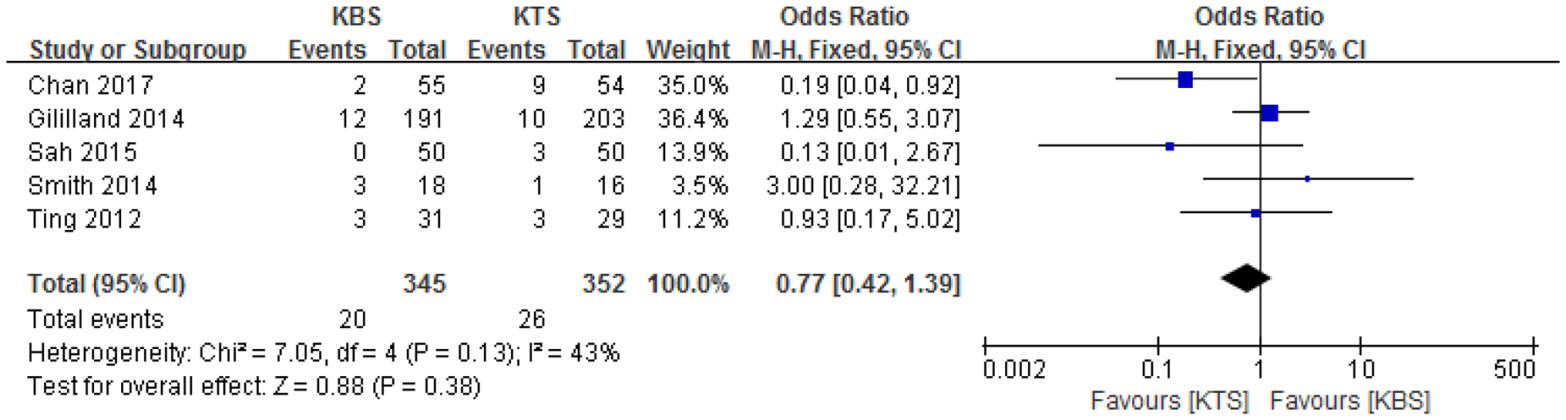
Forest plot and meta-analysis of complications

All five studies reported the complications [1, 2, 11, 13, 16]; subsequently, the data from these studies were pooled. Patients in both groups experienced similar rates of complications (OR 0.77, 95% CI 0.42–1.39, *P* = 0.38, *I*^2^ = 43%, *P* = 0.13; Fig. 4). Full details regarding complications are summarized in Table 3. There were no differences between the two groups in the rates of stitch abscess, dehiscence, cellulitis, peri-incisional erythema, or pulmonary embolism.

**Table 3:** Pooled outcomes of all the subgroups

Outcomes	No. of studies	No. of cases KBS/KTS	SMD/MD/OR	95% CI	Heterogeneity	*P* value of effect size
Wound suture time						
Arthrotomy	2	73/70	− 1.62	[− 2.00, 1.24]	*P* = 0.45; *I*^2^ = 0%	*Z* = 8.34 (P < 0.00001)
Subcutaneous	2	73/70	− 1.17	[− 2.07, 0.28]	*P* = 0.04; *I*^2^ = 76%	*Z* = 2.57 (*P* = 0.01)
Wound complications						
Stitch abscess	3	296/307	0.53	[0.20, 1.37]	*P* = 0.16; *I*^2^ = 45%	*Z* = 1.32 (*P* = 0.19)
Wound dehiscence	2	105/104	0.31	[0.03, 3.13]	NA	*Z* = 0.99 (*P* = 0.32)
Cellulitis	3	264/273	1.31	[0.48, 3.58]	*P* = 0.35; *I*^2^ = 4%	*Z* = 0.53 (*P* = 0.60)
Peri-incisional erythema	1	31/29	0.60	[0.09, 3.86]	NA	*Z* = 0.54 (*P* = 0.59)
Pulmonary embolism	1	191/203	3.20	[0.13, 79.15]	NA	*Z* = 0.71 (*P* = 0.48)
Knee range of motion						
≤ 6 weeks	2	105/104	− 0.74	[− 4.19, 2.71]	*P* = 0.50; *I*^2^ = 0%	*Z* = 0.42 (*P* = 0.67)
≤ 3 months	2	105/104	− 0.30	[− 2.62, 2.02]	*P* = 0.05; *I*^2^ = 75%	*Z* = 0.26 (*P* = 0.80)
Knee society score						
≤ 6 weeks	2	246/257	− 0.22	[− 3.10,2.66]	*P* = 0.37; *I*^2^ = 0%	*Z* = 0.15 (*P* = 0.88)
≤ 3 months	2	105/104	− 2.04	[− 3.92, 0.15]	*P* = 0.35; *I*^2^ = 0%	*Z* = 2.12 (*P* = 0.03)
1 year	1	50/50	− 0.5	[− 3.03, 2.03]	NA	*Z* = 0.39 (*P* = 0.70)
Intraoperative events						
Suture breakages	2	241/253	1.99	[0.01, 401.87]	*P* = 0.010; *I*^2^ = 85%	*Z* = 0.25 (*P* = 0.80)
Needle stick injuries	1	191/203	0.21	[0.02, 1.80]	NA	*Z* = 1.43 (*P* = 0.15)

*KBS* knotless barbed sutures, *KTS* knotted traditional sutures, *SMD* standardized mean difference, *MD* mean difference, *OR* odds ratio, *NA* not applicable

### Knee range of motion

**Fig. 5:**
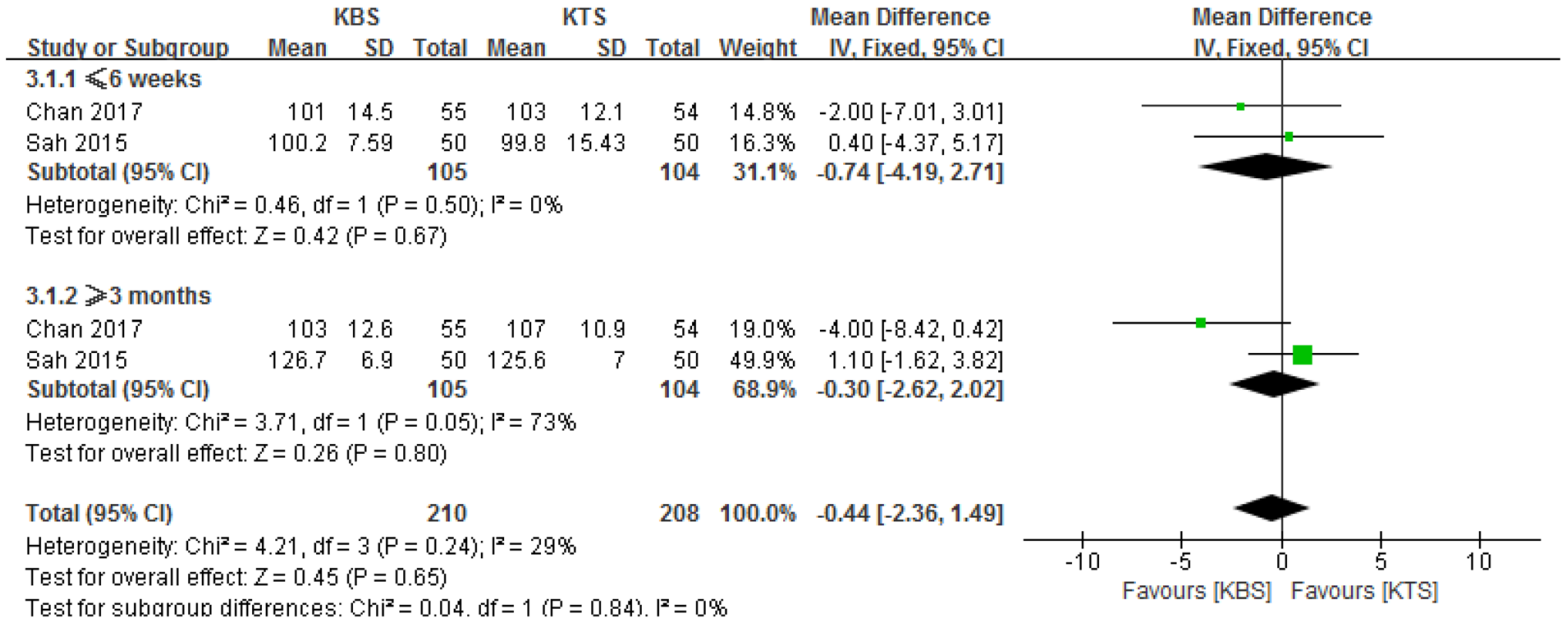
Forest plot and meta-analysis of knee range of motion

Two studies reported knee ROM at 6 weeks and 3 months after surgery [1, 2]. Therefore, we performed subgroup meta-analyses to compare the knee ROM based on the date. Full details for knee ROM are summarized in Table 3. There were no significant differences between the two groups at postoperative 6 weeks and 3 months (MD − 0.74, 95% CI − 4.19 to 2.71, *P* = 0.67, *I*^2^ = 0%, *P* = 0.50; MD − 0.30, 95% CI − 2.62 to 2.02, *P* = 0.80, *I*^2^ = 73%, *P* = 0.05; respectively; Fig. 5).

### Knee Society Score

**Fig. 6:**
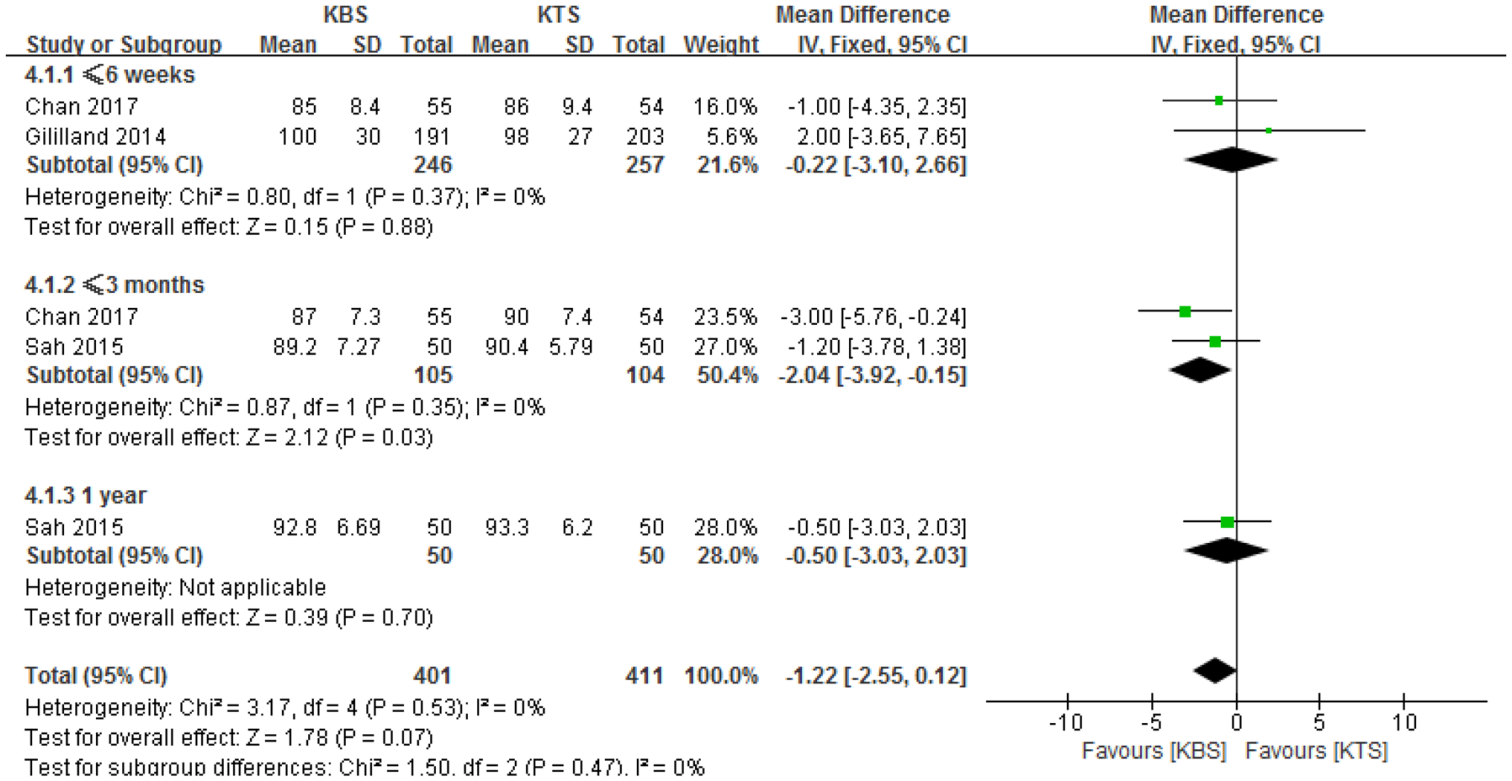
Forest plot and meta-analysis of Knee Society Score

Three studies reported the KSS after surgery [1, 2, 13]. Therefore, we performed subgroup meta-analyses to compare the KSS based on the date. Full details for the KSS are summarized in Table 3. There were no significant heterogeneities among the subgroups (*P* = 0.37, *I*^2^ = 0%; *P* = 0.35, *I*^2^ = 0%; respectively, Fig. 6). At postoperative 3 months, the KBS group obtained a higher KSS. However, there were no significant differences between the two groups at postoperative 6 weeks and 1 year.

### Wound closure cost

**Fig. 7:**
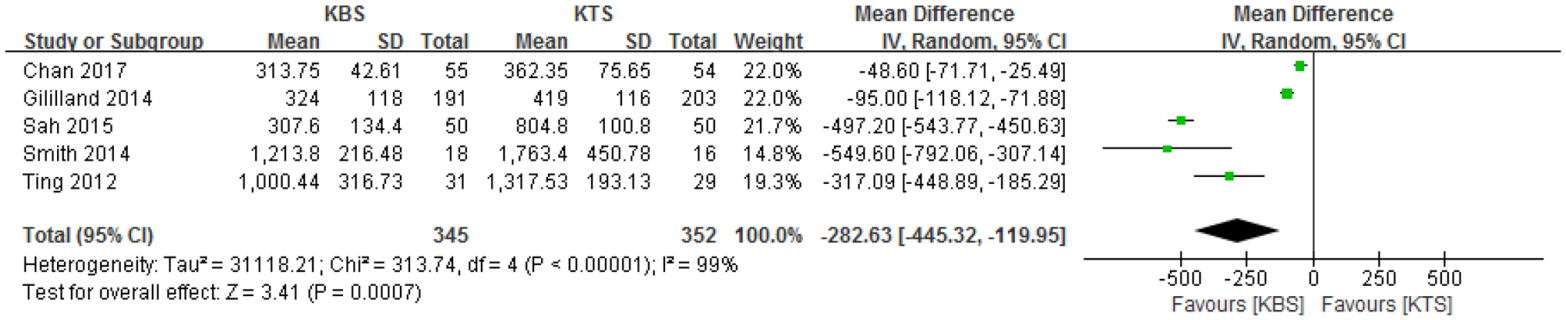
Forest plot and meta-analysis of cost

All five studies reported the wound closure cost [1, 2, 11, 13, 16]; subsequently, the data from these studies were pooled. The cost differences in terms of closure time in the operating room and materials between the two groups are summarized in Table 2. Analysis of the pooled cost data showed that KBS was associated with 282.63 USD lower costs than KTS (MD − 282.63, 95% CI − 445.32.00 to − 119.95, *P* = 0.0007, *I*^2^ = 99%, *P* < 0.00001, Fig. 7).

### Intraoperative events

**Fig. 8:**
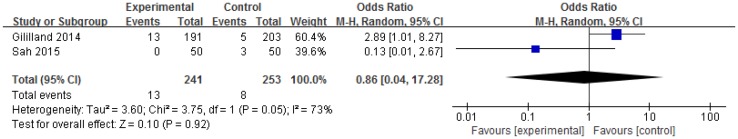
Forest plot and meta-analysis of intraoperative events

Data for intraoperative events were only available in two studies [2, 13], and the results showed high heterogeneity (*P* = 0.05, *I*^2^ = 73%). Therefore, we performed subgroup meta-analyses to compare the different intraoperative events (Table 3). The pooled results showed that there were no significant differences between the two groups in terms of intraoperative events (OR 0.86, 95% CI 0.04–17.28, *P* = 0.92; Fig. 8).

### Result of the subgroup analysis

Table 3 shows the results of the subgroup analysis. KBS significantly reduced the arthrotomy and subcutaneous wound closure times. No significant difference was observed in the risk of different complications. There were no significant differences between the two groups in postoperative knee ROM and KSS. However, the KBS obtained a higher KSS at postoperative 3 months. The incidence of intraoperative events did not differ significantly between the two groups.

## Discussion

To our knowledge, the risk that KBS use will increase the incidence of potential complications in TJA remains controversial. Our review incorporated 5 studies and 600 participants who were treated with primary TKA or THA. The purpose of our meta-analysis was to determine whether KBS could reduce wound closure times, costs, and potential complications. In this analysis of studies, we found that (1) KBS were not clearly associated with intraoperative events, postoperative functional recovery, or complications, and (2) KBS could reduce wound closure times and closure costs.

As reported in a previous study, KBS were able to reduce wound suturing time in our study [17–20]. Eickmann et al. found that KBS saved approximately 11.5 min in TKA compared with KTS [14]. Furthermore, in Mansour’s study of spinal fusion, KBS reduced the wound suture time by 40%. Theoretically, KBS are self-anchored and does not require a knot, thus allowing faster closure [12]. However, we performed a subgroup analysis, because the wound suture time showed high heterogeneity. The subgroup analysis showed that the suture times for different layers were better in the KBS group than in the KTS group despite the heterogeneity of the subcutaneous layer sutures. Because the surgical procedure differs for the treatment of joint incisions and shallow skin closures, a significant heterogeneity of wound suturing times is hard to prevent.

According to the previous studies, the use of KBS may lead to more postoperative complications compared with KTS [15, 21]. In general, the junction can cause uneven pressure on the soft tissue, leading to ischaemia, and large adsorption junctions may lead to local tissue inflammation and scar formation, which are potential risk factors for infection. Shermak found that KBS increased the risk of complications of arm wound healing, suggesting that the increase in surface area caused by barbs and continuous stitching results in the spread of inflammation along the length of the closure [22]. However, we observed no difference in the overall incidence of these complications between TJA surgeries with KBS and KTS, consistent with the findings of Zhang [20]. Taking into account the clinical differences among the different complications, we conducted a subgroup analysis; the results showed that the rates of different complications did not differ significantly between the KBS and KTS groups. This may be because KBS provides more uniform tissue tension, which reduces the risk of ischaemia, and a lack of nodules, which reduces the potential for local inflammatory response and extruded suture/sterile abscess, thereby reducing the risk of wound complications [13, 23].

Moreover, there were no differences in the incidence of intraoperative events between the KBS and KTS groups. The traditional stitching method requires interruptions for knotting, which increase the risk of suture breakages and needle stick injuries. In contrast, KBS allows continuous stitching and avoid the traditional knot, thereby reducing the rate of intraoperative events. There may be considerable heterogeneity in the intraoperative events associated with wound suturing due to the different surgical procedures used for KBS. In addition, in Chan’s studies, there were significantly more positive leak tests in the KTS group. A study of simulated tonic arthritis of the body found that, compared with KTS, the use of KBS closure decreased the leakage rate by 74% [10]. Due to the uniform distribution of the barbs over the entire suture, the breakage or loosening of a single suture ring does not easily cause the entire suture to fail. This is important for ensuring the watertight closure of joint incision wounds [10].

The quality of wound closure is critical to the high tension resistance of knee surgery wounds, especially for rapid recovery regimens. Our study found no significant difference in postoperative ROM values between patients who received KBS and those who received KTS in the early (< 6 weeks) and midterm (< 3 months) postoperative periods. This result is consistent with the previous reports that there is no significant difference between KBS and KTS in terms of the average degree of extension and flexion and the KSS score [12–14]. In the midterm (< 3 months), the patients who received KBS had a higher KSS than those who received KTS, and this difference was clinically unimportant. However, whether there is a difference in the long-term efficacy of KBS and KTS requires RCTs with long-term follow-up to determine the effect of suture type on functional recovery.

Our analysis found that KBS significantly reduced the cost of TJA surgery. Similarly, Zhang found that KBS could reduce wound closure time by 3.56 min and reduce costs by $290.72 compared with KTS [20]. These savings are comparable to those reported in this study; however, the cost savings are dependent on the operating costs in each region. The average cost of surgery in 100 US hospitals was $62 per minute (range $22–133 per minute) [24]. The estimated operating room costs are based on the average cost of the professionals and resources required for these operations. It is noteworthy that although KBS materials are more expensive than KTS materials, the shorter operating time leads to lower overall costs. Another implication of the time-saving nature of KBS is the reduced cost associated with its use. When cost-effectiveness is calculated, we should consider overall resource utilization. Currently, no studies have truly addressed the issue of cost in a way that is relevant to most institutions. In addition to cost savings, less time in the operating room reduces exposure to anaesthesia, which is safer for patients and helps to control healthcare costs. These potential benefits were not considered in the cost analysis.

To the best of our knowledge, this meta-analysis of the validity and safety of KBS and KTS includes all randomized-controlled trials. However, it has the following restrictions. Mainly because of the different levels of stitching, a variety of suture methods were used, and the stitching technique was not uniform; consequently, a significant heterogeneity in the wound suture time and total cost was inevitable. In addition, the included studies were less focused on evaluating hip function post-TKA. In all studies, the follow-up time was relatively short, which prevented an assessment of long-term efficacy, especially in terms of postoperative functional examinations. Finally, the limited number of studies, the small number of samples, the different protocols used, and the different backgrounds of the participants may weaken our analysis. Therefore, more high-quality research is needed in the future to determine the effectiveness and safety of KBS in TJA.
